# Using exome sequencing to decipher family history in a healthy individual: Comparison of pathogenic and population *MTM1* variants

**DOI:** 10.1002/mgg3.405

**Published:** 2018-07-25

**Authors:** Monica Penon, Hengameh Zahed, Victoria Berger, Irene Su, Joseph T. Shieh

**Affiliations:** ^1^ Department of Pediatrics Division of Medical Genetics University of California San Francisco San Francisco California; ^2^ Department of Obstetrics, Gynecology and Reproductive Science University of California San Francisco San Francisco California; ^3^ Institute for Human Genetics University of California San Francisco San Francisco California

**Keywords:** adult, carrier, exome, genomic, *MTM1*, myotubular myopathy, neonatal, population variation, sequencing healthy, X‐linked

## Abstract

**Background:**

When a family encounters the loss of a child early in life, extensive genetic testing of the affected neonate is sometimes not performed or not possible. However, the increasing availability of genomic sequencing may allow for direct application to families in cases where there is a condition inherited from parental gene(s). When neonatal testing is not possible, it is feasible to perform family testing as long as there is optimal interpretation of the genomic information. Here, we present an example of a healthy adult woman with a history of recurrent male neonatal losses due to severe respiratory distress who presented to Medical Genetics for evaluation. A family history of additional male neonatal loss was present, suggesting a potential inherited genetic etiology.

**Methods:**

Although there was no DNA available from the neonates, by performing exome sequencing on the healthy adult woman, we found a missense variant in *MTM1* as a potential candidate, which was deemed pathogenic based on multiple criteria including past report.

**Results:**

By performing an analysis of all known *MTM1*‐disease associated mutations and control population variation, we can also better infer the effects of missense variations on *MTM1*, as not all variants are truncating. *MTM1*‐X‐linked myotubular myopathy is a condition that leads to male perinatal respiratory failure and a high risk for early mortality.

**Conclusions:**

The application of genetic testing in the healthy population here highlights the broader utility of genomic sequencing in evaluating unexplained recurrent neonatal loss, especially when genetic testing is not available on the affected neonates.

## 1 INTRODUCTION

The applications of genomic sequencing are rapidly expanding. Although trio sequencing has a substantial diagnostic yield, affected individual‐only and duo sequencing are also effective when there is consanguinity or narrowed genomic regions for interpretation. The application of broad sequencing to apparently healthy individuals is also being considered (Linderman, Nielsen, & Green, 2016). Classical medical and genetic teaching emphasizes the importance of a family history in evaluation, and this may be important to consider even when sequencing is more broadly applied (Bennett, 2012). Here, we present the application of exome sequencing for the scenario where affected offspring are not available and clinical exome sequencing is applied to an unaffected parent. Exome sequencing has been successfully used to investigate fetal demise (Shamseldin, Swaid, & Alkuraya, 2013), however if fetal/neonatal loss is in the past or if DNA is not available, autopsy by proxy may be needed (Shamseldin et al., 2017). The asymptomatic parent sequencing approach has been very successful in identifying homozygous conditions primarily, however it can be applied to other scenarios as well.

## 2 MATERIALS AND METHODS

### 2.1 Ethical compliance

Procedures followed were in accordance with the ethical standards of the responsible committee on human experimentation (institutional and national) and with the Helsinki Declaration of 1975, as revised in 2008. Protocol and procedures employed were reviewed and approved by the human subjects review committee. Informed consent from the family was obtained for publication of this clinical report.

### 2.2 Clinical

**Figure 1:**
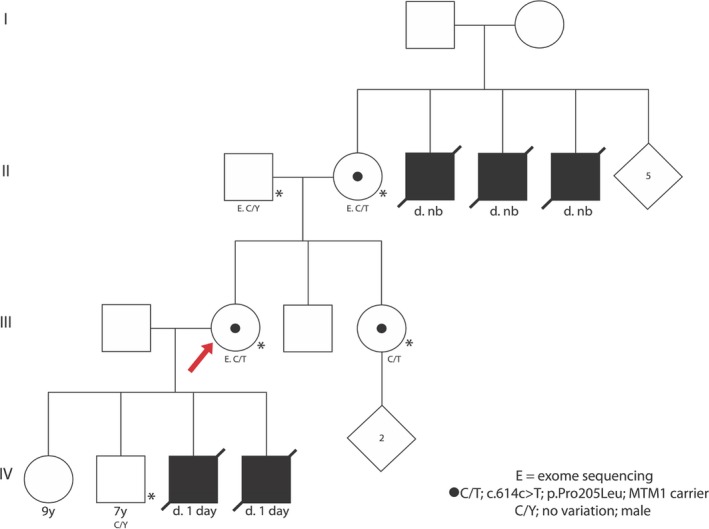
Family pedigree. An asterisk indicates genotyping was performed and E. indicates exome sequencing was performed. Shaded symbols indicate affected and carrier state

A 33‐year‐old healthy female with a history of recurrent male neonatal losses presented for genetic evaluation. She had lost two neonates early in life and was seeking evaluation to identify the etiology of her recurrent neonatal losses and assess the risk for future pregnancies. Her pregnancy history included two healthy children, a male and a female, born at term, but her first and fourth pregnancies, however, had resulted in male infants that died in the early neonatal period from severe respiratory distress and reported pulmonary hypoplasia at birth (Figure 1). The male fetus from her first pregnancy was born at 35–36 weeks of gestation with a weight of 2,150 g and length of 45 cm. He did not cry at birth. Resuscitation efforts were unsuccessful, and he passed away. An autopsy showed organ weights consistent with 35–36 weeks of gestation, except for a low combined lung weight (23 g; lung/body weight ratio of 0.0107 [reference 0.0255 ± 0.0027]), suggestive of isolated pulmonary hypoplasia. Furthermore, all organs, including the brain, were noted to have severe generalized acute congestion consistent with severe hypoxia. No evidence of infection or other congenital anomalies were noted. The male fetus from the fourth pregnancy also required resuscitation, was intubated at birth, but was apparently not able to breathe and passed away soon thereafter. That pregnancy was notable for a history of oligohydramnios that was mild at 29–30 weeks and severe at 38 weeks. No autopsy was performed. The patient and her husband were both of Hispanic ancestry and there was no known consanguinity. Prior to presentation to our clinic, the family had limited other evaluation. No genetic studies had been done on the infants that suffered demise. The patient reported that both she and her husband had karyotype studies that were normal. No archived tissue was available for testing from the infants that passed away.

### 2.3 Sequencing

Given the lack of available tissue from the affected, whole exome sequencing (WES) on the presenting healthy woman was pursued under the premise there may be an X‐linked condition. After completing the consent process with the patient and her parents, clinical trio exome sequencing was performed using capture with Agilent SureSelect Exome XT and sequencing with an Illumina HiSeq 2500 on blood samples. Alignment to reference and variant calling were performed as described (Lee et al., 2014). Mean coverage was 70× achieved in the targeted region of the human genome.

### 2.4 Variant analysis

Resulting variants were filtered by minor allele frequency (filtering out common variants >1%) and then assessed for known disease‐related genes. A primary gene list of 384 genes was also generated using the following keywords based on the clinical history obtained on the course of the neonates: Lung hypoplasia, pulmonary hypoplasia, hypoventilation, acinar hypoplasia, alveolar capillary dysplasia, oligohydramnios, small for gestational age, neonatal death, stillborn, sudden infant death, polyhydramnios. Keywords were used to search the Human Genome Mutation Database (HGMD) and OMIM for gene list generation. Furthermore, given the family pedigree with neonatal losses only in male offspring, we suspected an X‐linked inheritance pattern and therefore prioritized variants located on the X‐chromosome in analysis. X‐linked variants in a female could appear as heterozygous and were subject to an 0.1% MAF filter. Variants were assessed using de novo, X‐linked, recessive, and dominant inheritance patterns. A search for extended homozygosity was also performed. After filtering away common variants, the remaining variants were assessed by bioinformatics criteria including comparison to NHLBI ESP database, OMIM, HGMD, ExAC, and Clinvar and by conservation and functional prediction (Lee et al., 2014). A genomics board reviewed all possible variants of uncertain significance and potentially pathogenic/likely pathogenic variants.

### 2.5 MTM1 variants

To compare the *MTM1* variants from those with myotubular myopathy (OMIM 310400) to those from population controls, we aggregated pathogenic and likely pathogenic variants from NCBI Clinvar (Landrum et al., 2016) and the Leiden Open Variation Database (Fokkema et al., 2011) (https://www.ncbi.nlm.nih.gov/clinvar/?term=mtm1[gene], http://www.dmd.nl/nmdb2/home.php?select_db=MTM1) and all variants from ExAC (Lek et al., 2016) that were present in males or in both males and females. We used the reference sequence NC_000023.10, gene version NM_000252.2.

The resulting amino acid changes which corresponded to missense variant entries were examined for effect on the protein sequence. Since MTM1 has not been crystallized, to compare missense variant localization in cases of myotubular myopathy to those from ExAC, we examined the amino acid positions on the structure of MTMR2 (PDB 1ZSQ), a myotubularin‐family member similar to MTM1. Amino acids were highlighted on PDB 1ZSQ using the Protein Data Bank structure 3D view and using viewers such as JSmol. Missense variant locations were mapped to structural features, and variants were also classified by their amino acid effect. The localization to structural features was examined for both pathogenic variants and population variants, and these data were compared using Fisher's exact test.

## 3 RESULTS

Exome sequencing identified a total of 23,457 DNA variants in the patient, including 21,716 single nucleotide substitutions and 1,741 small deletions/insertions <10 bp. The patient was also found to have 8 rare homozygous, 9 compound heterozygous, and many rare heterozygous protein‐altering variants of uncertain clinical significance, however, all X‐chromosome variants were considered high priority given the family history. A heterozygous missense variant in the *MTM1* gene NC_000023.10:g.149809827C>T (chrX, hg19), NM_000252.2: c.614C>T; (p.Pro205Leu) was found, and it was maternally inherited. Variants in *MTM1* are associated with X‐linked myotubular myopathy (OMIM 310400), a congenital muscle disorder, which can cause severe congenital myopathy and early mortality (Laporte et al., 1996, 2000). The other variants were reviewed and based on variant‐reporting criteria, the *MTM1* variant was deemed highest potential. We assessed the potential pathogenicity of the *MTM1* variant using specific lines of evidence (Richards et al., 2015). (1) Past annotation of this variant has found it to be pathogenic (Biancalana et al., 2003; Oliveira et al., 2013; Tsai et al., 2005) and it was reported once in ClinVar, (2) Marked conservation of this variant across multiple species including fish, and (3) Functional studies support the importance of this amino acid residue (Taylor, Maehama, & Dixon, 2000). In the functional study, the *MTM1* variant was associated with a decrease in protein activity in yeast. No other variant was found that could explain the recurrent neonatal male losses. Variant segregation was also examined as the healthy son was also tested for the variant by targeted sequencing and did not have the variant. The proband's sister was also found to carry the variant. This variant was classified as pathogenic. Reports of this variant have found that it is typically associated with a severe form of the condition, although occasionally it is seen with mild/moderate forms (Biancalana et al., 2017; Tsai et al., 2005). With this variant, prenatal genetic counseling could be tailored for the planning of future pregnancies and for other family members.

**Figure 2:**
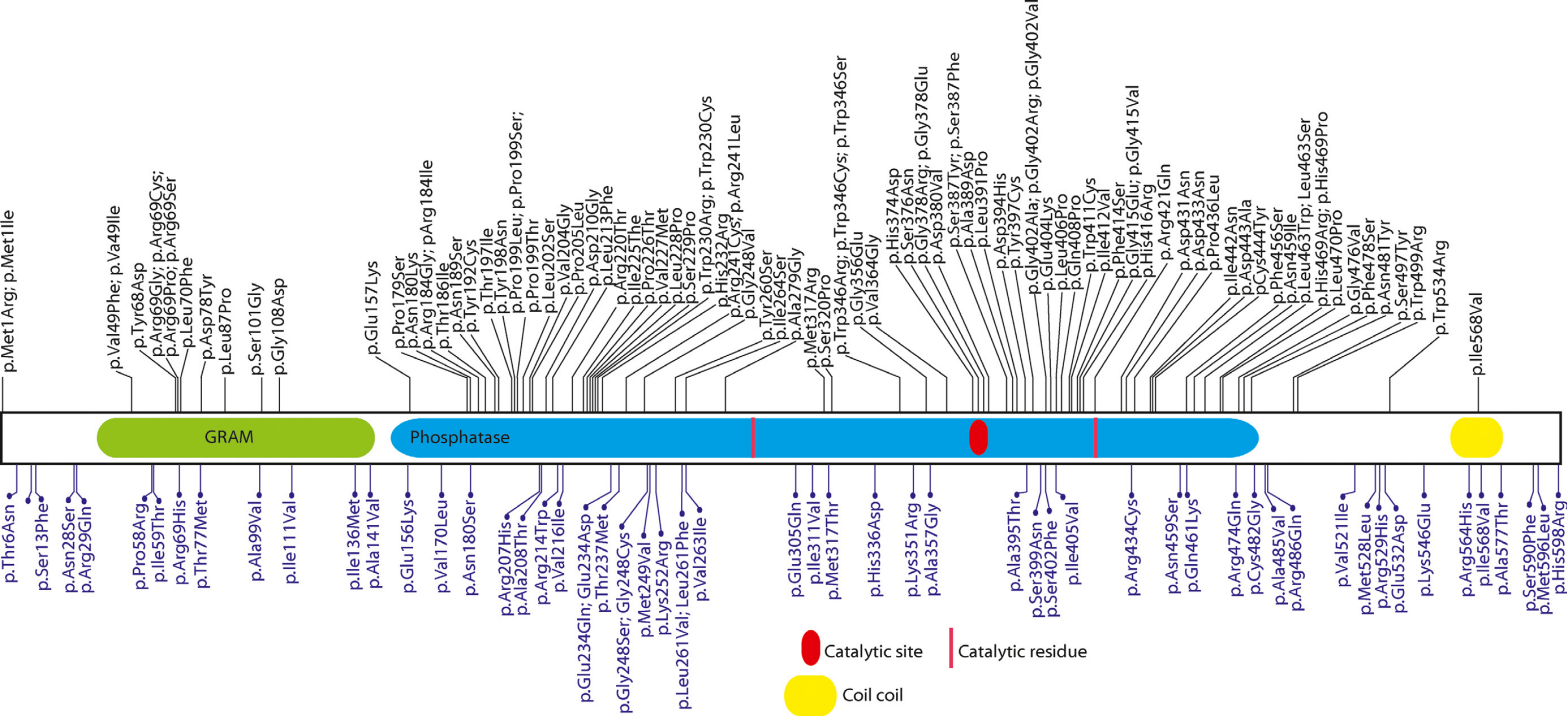
Comparison of variants in *MTM1*. The top are pathogenic/likely pathogenic variants and the bottom are population variants. MTM1 domains are shown, including GRAM, phosphatase, catalytic site and residues, and coiled coil domains. Reference sequence NC_000023.10, gene version NM_000252.2

**Figure 3:**
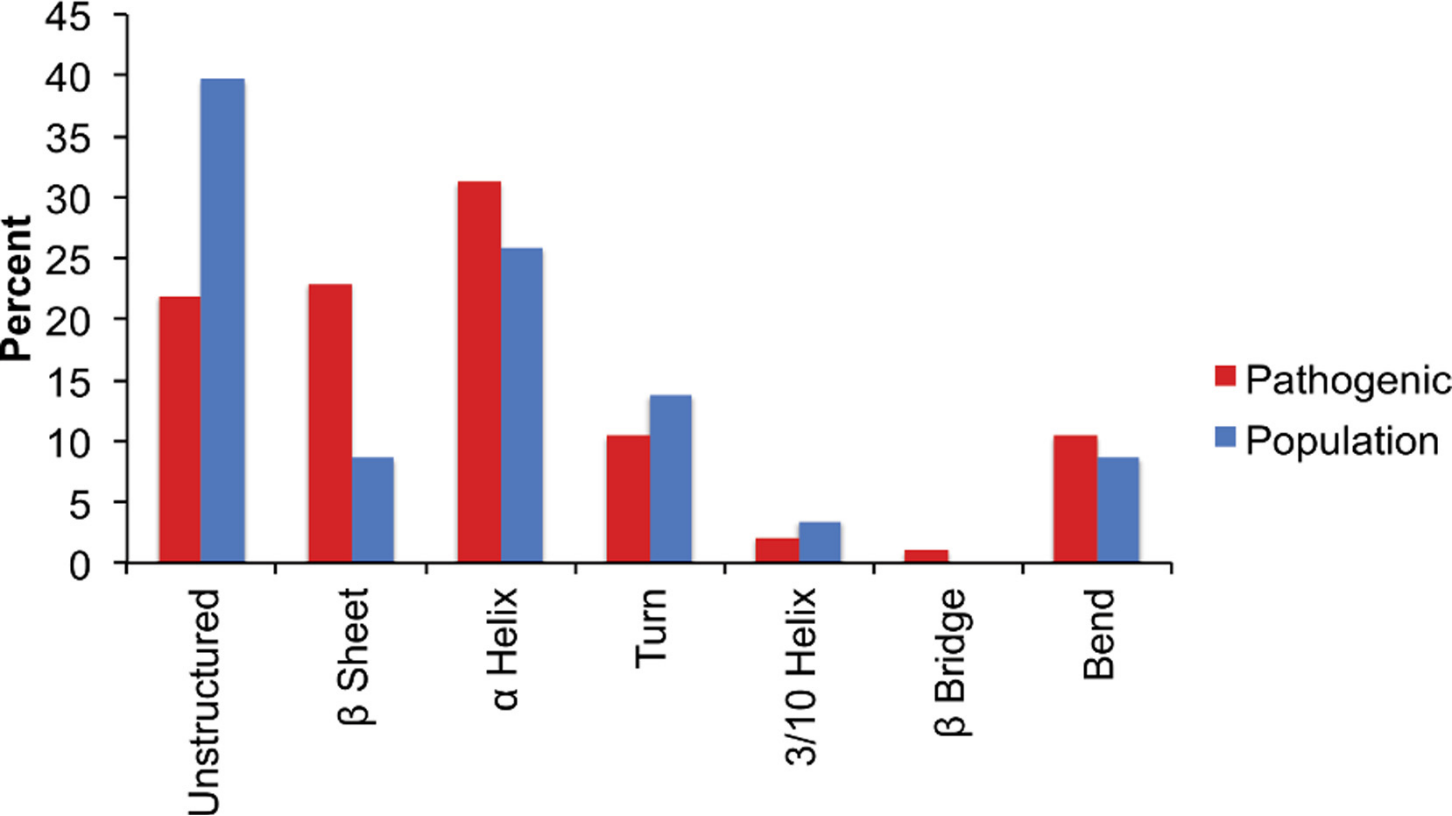
Percent of pathogenic and population variants found on different domains of the MTMR2 crystal structure. More pathogenic variants were found in beta sheet regions compared to population variants, while more population variants were found in areas of the protein outside of structural elements compared to pathogenic variants (Fisher's exact test, *p* < .05)

To examine the *MTM1* variants further, we compared all disease‐associated variants in *MTM1* to population variation in *MTM1* and examined their distribution along the coding sequence. To do this, we collected all *MTM1* variants from Clinvar and the Leiden Open Variation databases and examined likely pathogenic and pathogenic variants and all ExAC variants that have been found in the gene. *MTM1* disease variants can be high‐confidence loss‐of‐function alleles given truncation, splice‐site variants, or larger alterations. However, *MTM1* missense variation, regardless of pathogenicity, could occur throughout the gene length, and prediction of deleteriousness could be more difficult. We compared all the annotated pathogenic and likely pathogenic missense variants to all the control variants from males in ExAC, who would be unlikely to have severe *MTM1*‐associated disease. The disease‐associated variants seemed to have a greater concentration in the select domains of MTM1 (Figure 2), including the Pleckstrin Homology‐Glucosyltransferase Rab‐like GTPase Activator and Myotubularins (PH‐GRAM) domain, the myotubularin phosphatase domain, and the coiled coil region. When examined on the homologous myotubularin gene family model PDB 1ZSQ, variants involving the PH‐GRAM domain are easily distinguished from those in the myotubularin phosphatase domain, as expected (Figure S1). Interestingly, MTM1 and the related MTMR2 have an amino acid identity of 63%, however when we examined the pathogenic amino acid changes in MTM1, 95% of these changes occurred in amino acids conserved between MTM1 and MTMR2. When we examined the predilection of variants to occur in specific secondary structural features of the protein, disease‐associated variants were more commonly found in beta sheet‐associated amino acid residues whereas non‐disease‐associated variation were more commonly found in areas of the protein outside of secondary structure elements (*p* < .05, Fisher's exact test; Figure 3 and Table S1). These data might suggest that the disease missense variants may affect MTM1 in several ways including alteration of the conserved MTM1 domains, direct alteration of catalytic residues, or more direct loss of function.

## 4 DISCUSSION

Genomic sequencing plays an important role in undiagnosed diseases, but how should broad genomic sequencing be applied to asymptomatic individuals? Here the family history suggested the need for testing, but no affected individuals were available. Exome sequencing was offered to the asymptomatic family member, and the sequencing results provided diagnostic carrier status information for the woman. Subsequently, carrier information for extended family members was provided. Such results can have an impact on reproductive planning and emphasize the importance of select application of broad sequencing. As genome‐scale sequencing is increasingly applied to different clinical scenarios, it will be important to determine what genes in the exome should be assessed given the clinical context of the test.

Interestingly, several common commercial carrier‐testing companies did not include *MTM1* in their carrier testing panels until recently. Now that *MTM1* will be further tested, the data may lead to more variants that are yet unclassified for their significance. Our *MTM1* analyses here suggest features that help distinguish variants that are more likely to be pathogenic. Previous studies on *MTM1* mutations (Longo, Russo, Novelli, Sangiuolo, & D'Apice, 2016) found that missense variants were the most common type of variants in X‐linked myotubular myopathy cases, and they also assessed these based on functional prediction software and conservation. Our data support the importance of conserved MTM1 residues, as 95% of the missense variants reported were conserved between MTM1 and MTMR2. Protein structure and protein domain information could be further incorporated to optimize prediction algorithms (Ge, Kwok, & Shieh, 2015; Ge et al., 2016). Also, further variant curation from affected individuals (Biancalana et al., 2017) and from the population should also allow for better data on variant pathogenicity. X‐linked myotubular myopathy may also be under‐diagnosed in natal and carrier testing. Importantly, there is a clinical spectrum to those with *MTM1*‐myotubular myopathy, and further work into early detection and clinical trials will be important (Danièle et al., 2018; Raess et al., 2017).

As sequencing becomes more available, more asymptomatic individuals will seek broad genetic information about themselves in the future. The most critical clinical genomic information likely depends on our understanding of medically relevant genes and their actionability. Efforts such as ClinGen are annotating important disease genes, and these gene lists now include x‐linked myotubular myopathy, among many others (Ceyhan‐Birsoy et al., 2017). Which targeted critical gene list to focus on likely depends on the clinical context of the sequencing. The specific genes applicable for returning to individuals may go well beyond the current ACMG recommended genes. In particular, sequence interpretation for asymptomatic individuals should likely follow clear guidelines on what genes are being assessed and what types of variants are most important.

## CONFLICTS OF INTEREST

The authors have no conflicts of interest to declare regarding this manuscript.
