# Supplementary figures and images for: Using exome sequencing to decipher family history in a healthy individual: Comparison of pathogenic and population MTM1 variants
Source: Mol Genet Genomic Med. 2018 Jul 25;6(5):722–7. doi: 10.1002/mgg3.405 (PMC6160706; doi:10.1002/mgg3.405)

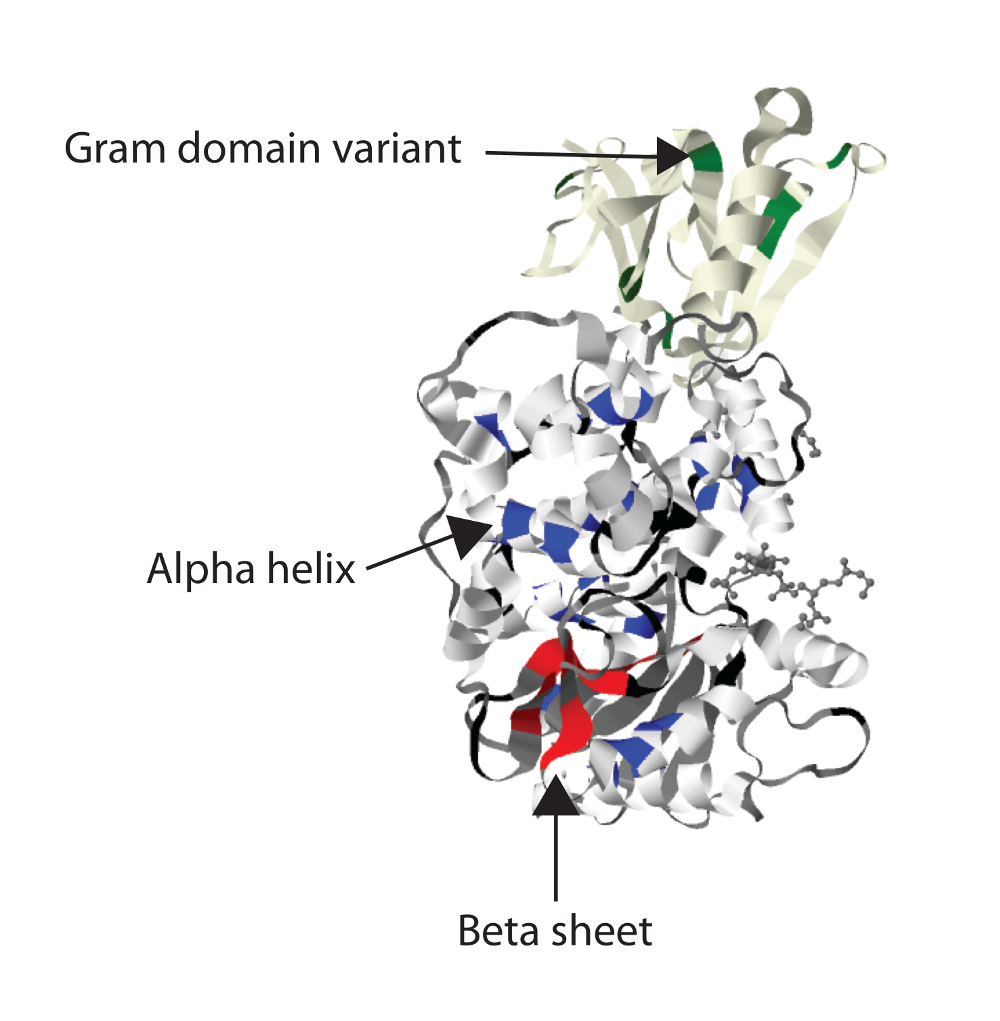

Supplement: Supplementary file 1 [file MGG3-6-722-s001.tiff]
